# Impact Mechanism of the Ecological Vulnerability of Highly Developed Islands Based on the Bayesian Network Model—Applied to the Changshan Islands

**DOI:** 10.3390/ijerph18084150

**Published:** 2021-04-14

**Authors:** Keyu Qin, Haijun Huang, Jingya Liu, Liwen Yan, Yanxia Liu, Haibo Bi, Zehua Zhang, Yi Zhang

**Affiliations:** 1 Key Laboratory of Marine Geology and Environment, Institute of Oceanology, Chinese Academy of Sciences, Qingdao 266071, China; kyqin@qdio.ac.cn (K.Q.); hjhuang@qdio.ac.cn (H.H.); yanliwen@qdio.ac.cn (L.Y.); liuyanxia@qdio.ac.cn (Y.L.); bhb@qdio.ac.cn (H.B.); zzh@qdio.ac.cn (Z.Z.); zhangyi1@qdio.ac.cn (Y.Z.); 2 University of Chinese Academy of Sciences, Beijing 100049, China; 3 Center for Ocean Mega-Science, Chinese Academy of Sciences, Qingdao 266071, China; 4 Laboratory for Marine Geology, Qingdao National Laboratory for Marine Science and Technology, Qingdao 266061, China; 5 Institute of Geographic Sciences and Natural Resources Research, Chinese Academy of Sciences, Beijing 100101, China; 6 College of Ocean Science and Engineering, Shandong University of Science and Technology, Qingdao 266000, China

**Keywords:** island, ecological vulnerability, Bayesian network, impact mechanism, the Changshan Islands

## Abstract

Islands are one of the most sensitive interfaces between global changes and land and sea dynamic effects, with high sensitivity and low stability. Therefore, under the dynamic coupling effect of human activities and frequent natural disasters, the vulnerability of the ecological environment of islands shows the characteristics of complexity and diversity. For the protection of island ecosystems, a system for the assessment of island ecosystems and studies on the mechanism of island ecological vulnerability are highly crucial. In this study, the North and South Changshan Islands of China were selected as the study area. Considering various impact factors of island ecological vulnerability, the geographical information systems (GIS) spatial analysis, field surveys, data sampling were used to evaluate island ecological vulnerability. The Bayesian network model was used to explore the impact mechanism of ecological vulnerability. The results showed that the ecological vulnerability of the North Changshan Island is higher than that of the South Changshan Island. Among all the indicators, the proportion of net primary productivity (NPP) and the steep slope has the strongest correlation with ecological vulnerability. This study can be used as references in the relevant departments to formulate management policies and promote the sustainable development of islands and their surrounding waters

## 1. Introduction

According to the modern international marine legal system, islands, together with internal waters, territorial waters, exclusive economic zones, and continental shelves, constitute the ‘marine territory’ of coastal countries. Islands play an irreplaceable role in safeguarding marine rights and interests and national security, resisting natural disasters, and ensuring the safety of the marine ecological environment. Simultaneously, islands are important for enhancing the marine economy and expanding the development space [1]. The type and regional structure of island landforms are relatively simple, the biodiversity index and stability of ecosystems are poor. The island ecological environment coupled with the interactions of oceans, land, gas, global changes, and disturbances caused by human activities becomes vulnerable, which renders the island a sensitive zone and typical ecological vulnerable zone (ecotone) with frequent disasters [2]. However, because of island independence, the sensitivity and self-regulation capacity of the islands are considerably different from those of the land [3]. Therefore, islands have become an important topic for scholars to explore ecosystem vulnerability under the interference of human and natural factors [3,4].

Reasonable assessment of ecological vulnerability is important for the protection of island ecosystems [5,6]. Studies on island ecological vulnerability have mainly focused on the impact of natural factors on ecosystems [7], such as the effect of hurricanes on natural ecosystem and the influence of humans on animals and plants living in the island [8]. With an increase in climate-change studies, scholars have paid considerable attention to sea-level rise [9]. Climate change and sea-level rise have become the most sought-after study topics. In the climate change background, island ecosystem feedback has emerged as a new research direction [3,10]. With the amplification of studies, scholars have focused on the impact of not only natural factors but also social and economic factors on ecosystems [11,12]. Some researchers have explored the mutual feedback relationship between human activities and island ecosystems [13]. Some scholars have paid attention to people’s perception, by using psychological and histological theories to study ecosystem vulnerability. In addition, some scholars have studied the vulnerability of agricultural ecosystems, have carefully classified the vulnerability of island ecosystems, and have proposed corresponding policy recommendations. For example, Mandel et al. evaluated the vulnerability of climate changes towards agricultural ecosystems in small islands by considering Indian islands as an example [14]. Currently, although island ecological vulnerability is assessed by integrating natural and human factors, studies have mainly focused on the assessment of ecological vulnerability of specific islands, and a universal system for island ecological vulnerability assessment remains lacking. Island ecological vulnerability must be further explored.

In recent years, the increased intensity of island development, the highly diversified methods of development and island resource utilisation [15], and the relative lag of island management policy and supervision mechanism have led to several adverse effects on the ecological management and sustainable development of islands [16,17,18]. Many factors affect island ecological vulnerability. However, the identification of the coupling relationship between influencing factors and island ecological vulnerability is difficult. Moreover, identifying the importance of influencing factor ranking in inducing island ecological vulnerability is difficult [19,20,21]. Some scholars have studied the mechanism of the impact of ecological vulnerability on regional scales in detail by conducting spatial analysis through GIS technology and by exploring the changes in regional ecological vulnerability inducers for a long term [22,23]. However, the study progress on island ecological vulnerability is slow because of the particularity and complexity of the island geographical environment. A lack of understanding of the internal mechanism of island ecological vulnerability and the imperfection of assessment methods lead to the absence of island management, which results in new uncertainty risks to fragile island ecosystems. Therefore, the analyses of the impact mechanism of island ecological vulnerability play a highly important role in island ecosystem protection.

Therefore, aiming at outstanding issues such as the independence of island ecosystems, high sensitivity, low adaptability, and lagging related studies, the theoretical system and impact mechanism of island ecological vulnerability must be assessed to understand the effects of natural and human activities on island ecosystem and to obtain a scientific basis for balancing island ecosystem protection and economic development. This study analysed numerous indicators having an important impact on island ecological vulnerability. Combined with field surveys, data collection, literature summary, and data processing, the evaluation index system, and universal evaluation method that consider different impact factors were established. Considering the North and South Changshan Island as examples, ecological vulnerability was evaluated, and the main influencing factors and conditional probabilities of each influencing factor were identified using the Bayesian network model and entropy difference algorithm. This paper is innovative in ecological vulnerability assessment and provides a new perspective in the analysis of impact mechanism. The assessment of island ecological vulnerability and the study of impact mechanism, by considering the island ecosystem as a whole can provide technical and methodological support for related studies, provide scientific references for island integrated management, and promote the protection and sustainable development of island ecosystems.

## 2. Materials and Methods

### 2.1. Study Area

**Figure 1:**
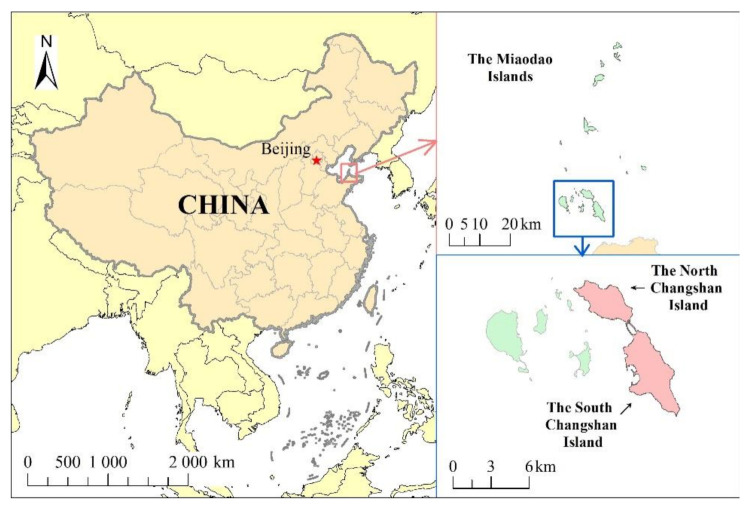
Study area.

The Changshan Islands, also known as the Miaodao Islands, are located in the connecting belt of the Bohai Rim Economic Circle, with South Korea and Japan to their east. The islands are distributed in the SSW-NNE direction at the confluence of the Yellow and Bohai Seas between the Shandong and Liaodong Peninsulas, which guard the Bohai Strait, and are the maritime gateway of the Beijing—Tianjin—Tangshan metropolis [24] (Figure 1). This study focused on the largest island of the Changshan Islands, the South Changshan Island, the North Changshan Island situated in the north, and their surrounding waters. Moreover, the South Changshan Island is the largest island in Shandong and is under the jurisdiction of the Changdao County, Yantai City, with 18,786 permanent residents (2015). It is connected to the North Changshan Island through an artificial dike in the north. The North Changshan Island has a nearly elliptical shape with a land area of 7.98 km^2^ and a coastline of 15.41 km, respectively. Currently, 2614 permanent residents live in the North Changshan Island [25].

An island has clear boundaries, and these boundaries can be easily assessed. The determination of their surrounding waters is complicated. The commonly used methods for this determination are as follows: (1) based on the island coastline, use the area extending 12 nautical miles outward as the surrounding sea range area; (2) refer to coastal wetland delineation. The study scope is the −6 m shallow sea area around the island; (3) for islands with residents, determine the area around the island according to the main impact area of the island’s human activities. Various appropriate methods can be selected for different islands to determine their surrounding sea areas according to their actual conditions. This study considered the influence of the factors of the Changshan Islands in the north and south on the sea area and the impact of the island land on the surrounding sea area and referred to the literature. The 1 km out extension of the island land was used as the assessment scope of the final result of the ecological vulnerability of the South and north Changshan Islands.

### 2.2. Research Structure

**Figure 2:**
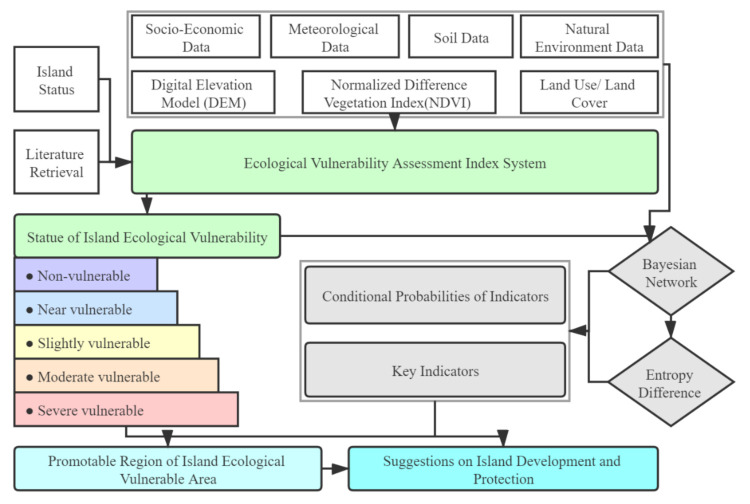
Technology roadmap.

This study can be divided into 4 steps: first, collect and collate the natural and socio-economic data of the Changshan islands; second, develop the island ecological vulnerability assessment index system to determine the island’s ecological vulnerability, according to the actual case of the island and literature reference; then, analyse the island ecological vulnerability assessment indicators and results and identify the key indicators affecting island ecological vulnerability based on the Bayesian network model and entropy difference method; Finally, identify the areas that can be promoted, and propose suggestions for scientific development and ecological protection (Figure 2).

### 2.3. Ecological Vulnerability Assessment

In the third report of the Intergovernmental Panel on Climate Change (IPCC), ecological vulnerability is defined as the combination of external interferences (exposure), sensitivity to external interferences (sensitivity), and capacity to adapt to external interferences (adaptability) [26]. According to this definition, an Exposure-Sensitivity-Adaptability (E-S-A) framework is proposed [27,28]. The E-S-A framework has been modified and successfully applied to study the ecological vulnerability of islands in China [16,29]. Therefore, this paper continues to use the E-S-A framework as an extension of the ecological vulnerability of islands through emphasis of the interference factors of human activities on the island.

According to the principle for selection of ecological vulnerability assessment indicators for high-intensity development islands, this study selected these indicators by referencing relevant domestic and international ecological evaluation literature and consulting experts from the island ecology field. To evaluate the ecological vulnerability of the islands, 24 indicators were selected (Table 1). Although the ecological vulnerability evaluation of islands is comprehensive, the Delphi method was used to provide subjective weights to each indicator. By combining experts’ professional knowledge, experience, and numerous questionnaires, a consultation form was issued to 76 experts from the field of island ecology in China’s universities and research institutes, and finally, 53 valid questionnaires were collected. The possible impact of each indicator on ecological vulnerability was divided into 5 levels with different grades (slight: 1, slight-medium: 2, medium: 3, medium-high: 4, high: 5) in the consultation form. By calculating the proportion of the total score of each indicator in the total score of the element layer, the weight of each indicator was obtained.

The index system of the island ecological vulnerability assessment constituted three layers, target, element, and index layers, which include three objectives, eight elements, and 23 indicators. The target layer comprises the exposure, sensitivity, and adaptability of island ecological vulnerability. The element layer comprises eight elements: B1 natural pressure, B2 human disturbances, B3 ecological conditions, B4 environmental conditions, B5 self-regulation, B6 social support conditions, B7 environmental protection, and B8 integrated management level.

#### 2.3.1. Assessment of the Island Ecological Vulnerability Single Factors

The single-factor index of island ecological vulnerability can be obtained as follows:

(1) $$RC_{i}=\left(\begin{array}{ll} \frac{C_{i}}{S_{i}}, & when\;C_{i}\;\mathrm{is}\;a\;\mathrm{negative}\;\mathrm{indicator}\\ \frac{S_{i}}{C_{i}}, & when\;C_{i}\;\mathrm{is}\;a\;\mathrm{positive}\;\mathrm{indicator} \end{array}\right)$$

where $RC_{i}$ represents the assessment result of indicator *i*, $C_{i}$ is the index value, and $S_{i}$ is the index standard value.

#### 2.3.2. Assessment of Island Ecological Vulnerability Elements

Based on the evaluation results of each index, different elements were calculated. The calculation formula is as follows:

(2) $$RB_{x}=\overset{n}{\underset{i=1}{\sum }}RC_{i}\times W_{i}$$

where *RB_x_* is the evaluation result of element *i*, and *W_i_* is the weight value of the index *i* in element *x* (the sum of weights of each index in a single element is 1).

#### 2.3.3. Sub-Objective Assessment of Island Ecological Vulnerability

Based on the evaluation results of each factor, the three objectives were calculated. To ensure the comparability of ecological vulnerability assessment in different study areas, the equal weight method was used for the sub-objective assessment. The calculation formulae used are as follows:

(3) $$RE=\frac{1}{2}\left(RB_{1}+RB_{2}\right)$$

(4) $$RS=\frac{1}{2}\left(RB_{3}+RB_{4}\right)$$

(5) $$RA=\frac{1}{4}\left(RB_{5}+RB_{6}+RB_{7}+RB_{8}\right)$$

where *RE*, *RS*, and *RA* are the evaluation results of exposure, sensitivity, and adaptability, respectively.

#### 2.3.4. Assessment of the Island Ecological Vulnerability Index (IEVI)

The *IEVI* can be calculated using the three objectives. The calculation formula and classification levels are as follows (Table 2). The *IEVI* is divided into 5 levels according to IPCC’s classification [26], and their respective confidence intervals are modified in the paper (based on the confidence range of the environment carrying capacity of State Oceanic Administration PRC [30] and the collective judgement of the author’s observational evidence and island features): non-vulnerable (*IEVI* ≤ 0.6), moderately near vulnerable (0.6 < *IEVI* ≤ 0.7), slightly vulnerable (0.7 < *IEVI* ≤ 0.9), moderately vulnerable (0.9 < *IEVI* ≤ 1.0), and severely vulnerable (*IEVI* > 1.0).

(6) $$IEVI=\sqrt[{3}]{RE\times RS\times RA}$$

### 2.4. Conditional State Subset Recognition

**Figure 3:**
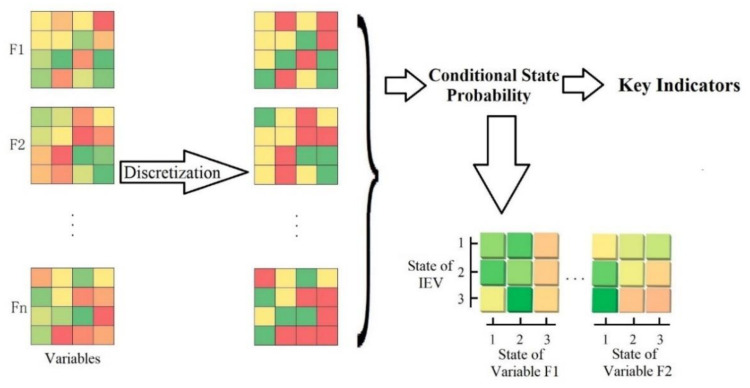
Spatial promotion based on the Bayesian network model.

In this study, the Bayesian network model was used to analyze the ecological vulnerability mechanism. The Bayesian network is expressed as a conditional probability table, which constitutes two parts: structure and parameter learning [31,32,33,34,35]. The data prepared were raster data, which were recorded as X, and X = {X_1_, X_2_, ···, X_n_}, where X_1_, X_2_, ···, X_n_ represent n evaluation indicators. Each index was expressed as a different array. Each index Xi exhibited mi States, which were recorded as Xi = {X_i,1_, X_i,2_, ···, X_i,mi_}, where X_i,1_, X_i,2_, ···, X_i,mi_ are different states of variable Xi [36,37]. By calculating the conditional probabilities of different indicators in various states and other indicators in different states, a conditional matrix was constructed [38]. According to the conditional probability calculated using Python programing, the conditional probability between pairs was expressed in the probability graph form. The conditional and promotable state subsets were analyzed by constructing a conditional probability graph (Figure 3).

### 2.5. Identification of Key Indicators

Selecting the index having the highest impact on ecological vulnerability is considerably important for promotable state subset evaluation and further spatial promotion. The index with the highest impact on ecological vulnerability is called the key indicator. The entropy difference method was used to calculate the entropy difference degree of each index for ecological vulnerability, which was used as the basis for key indicator selection [39,40].

Information entropy is usually used to evaluate the uncertain state of things, and the relationship between objects and backgrounds is generally discussed using the edge [41]. According to the information entropy definition, we calculated information entropy and entropy difference to evaluate the key indicators. The specific formula is as follows:

(7) $$V\left(I\right)=\left|S\left(E\right)-S\left(EI\right)\right|$$

where *V*(*I*) represents the entropy difference degree of each index with a value range from 0 to 1. *E* represents the ecological vulnerability node, *EI* denotes the index node affecting the ecological vulnerability, *S*(*E*) represents the entropy of ecological vulnerability, and *S*(*EI*) is the entropy of ecological vulnerability and joint occurrence of each index. The larger is *V*(*I*), the smaller is the impact of the index on the final ecological vulnerability; by contrast, the smaller is *V*(*I*), the larger is the impact of the index on the final ecological vulnerability. The specific calculation formulas for *S*(*E*) and *S*(*EI*) are as follows:

(8) $$S\left(E\right)=-\overset{n}{\underset{i=1}{\sum }}P\left(i\right)\log_{2}P\left(i\right)$$

(9) $$S\left(EI\right)=-\overset{n}{\underset{e=1,i=1}{\sum }}P\left(e,i\right)\log_{2}\left[P\left(e,i\right)\right]$$

where *P*(*e*) is the probability of island ecological vulnerability occurring in state *e*, *P*(*i*) is the probability of the index occurring in state *i*, and *P*(*e*, *i*) is the joint probability of island ecological vulnerability occurring in state e and the index occurring in state *i*.

### 2.6. Data Sources

Collect and segregate the existing data, especially the island survey and study results, and systematically analyze and assimilate the collected data (Table 3). The data included: (1) field survey basic layer; (2) literature collection and collation; and (3) remote sensing image preparation.

## 3. Results

### 3.1. Exposure Assessment

Natural pressure mainly considers the impact of typical natural disasters, the change rate of island area, the change rate of island shoreline and the proportion of steep slope area. Human interference mainly includes the population density of residents, the tourism population pressure, the impact of typical man-made environmental disturbance, the impact of land development, the impact of shoreline development and the impact of surrounding sea area development. The highest and lowest natural pressures of Changshan islands are 1.96 and 1.29, respectively. The natural pressure of the land area is high value, and that of the sea area is a low value, indicating that the land part is greatly affected by the natural pressure, and the sea area is relatively less affected by the natural pressure. The highest and lowest values of human interference in Changshan islands are 1.04 and 0.74, respectively. Certain areas near the coastline and sea of the North Island and South Changshan Island have high values and are greatly interfered by human activities. According to the comparison of the evaluation results of element layers with the distribution trends of various indicators, the proportion of the steep slope area has the greatest impact on natural pressure, and the development intensity of land and surrounding sea areas has the greatest impact on human interference.

**Figure 4:**
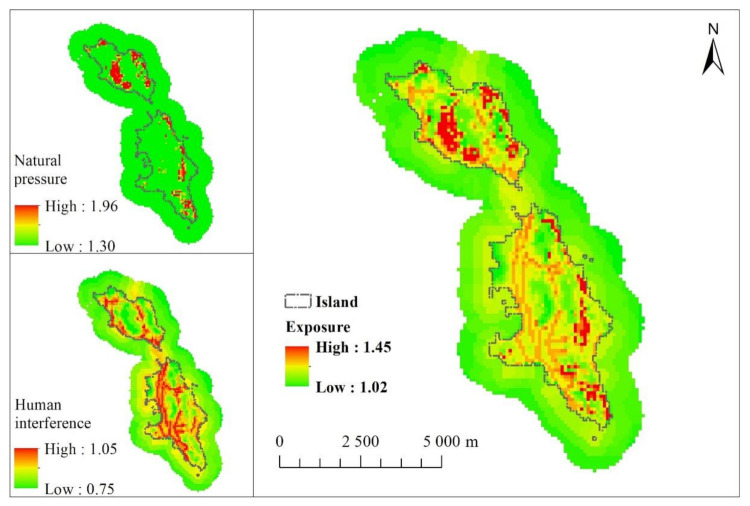
Exposure.

Figure 4 also presents the distribution map of the exposure degree of the Changshan Islands; the highest and lowest values are 1.45 and 1.02, respectively, and the regional difference is large. High-value areas are located along the coastline, indicating that the area is affected by human factors, with a high exposure and poor ecological environment. Compared with the island land, most of the sea area is the low-value area, indicating that the exposure degree of the sea area is lower. The island development activities of island residents, land and sea area development, have the most significant impact on the island’s ecological vulnerability. The influence of external interference on the sea area is lower than that on the island land.

### 3.2. Sensitivity Assessment

The difference in the ecological status between the sea and land areas is large. The coastal area of the Changshan islands is within a low value; hence, the ecological status does not easily affect by the external environment; the middle part of Changshan islands is within a high value; thus, the ecological status is relatively more vulnerable to the external influence. The highest and lowest environmental conditions of Changshan islands are 0.25 and 0.19, respectively. The environmental condition of the North Changshan Islands is worse than that of the South Changshan Island.

**Figure 5:**
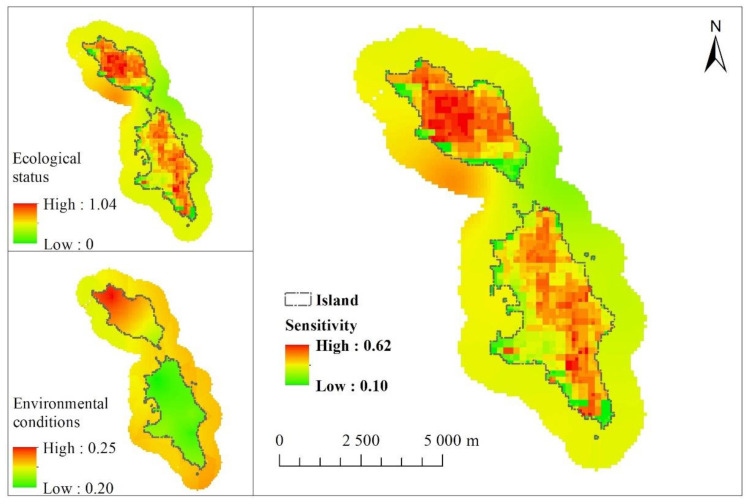
Sensitivity.

Figure 5 also presents the sensitivity distribution map of the Changshan islands; the highest and lowest values are 0.62 and 0.10, respectively, and the regional difference is large. The sensitivity distribution difference in the land is particularly large. The middle and coastal parts of the land area are the high- and low-value areas, respectively. The difference showed that the sensitivity of the middle part of the island is higher than that of the coastal area. The ecological environment of the middle part of the island is more likely to be affected after being disturbed and destroyed, indicating that the ecological sensitivity of this region is high.

### 3.3. Adaptability Assessment.

The self-regulation ability of the Changshan islands is 0.18, the social support condition is 0.97, the environmental protection is 0.98, and the comprehensive management level is 0.40. Because the adaptability indicators of Changshan islands are consistent, the evaluation results of each indicator are unique.

**Figure 6:**
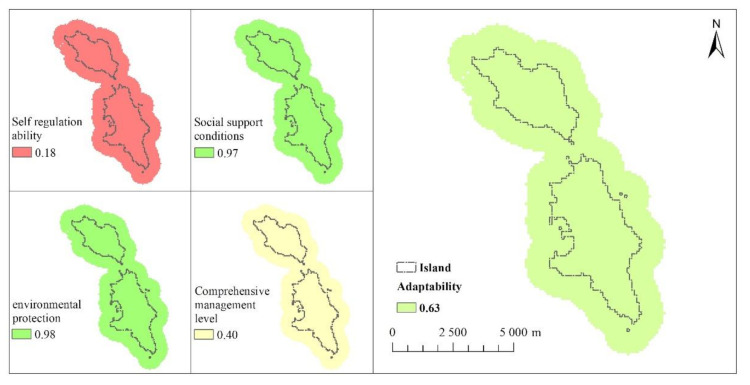
Adaptability.

Figure 6 presents the distribution map of adaptability of the Changshan Island; its unique value is 0.63. The adaptability of the whole study area is consistent.

### 3.4. Integrated Assessment of Island Ecological Vulnerability

**Figure 7:**
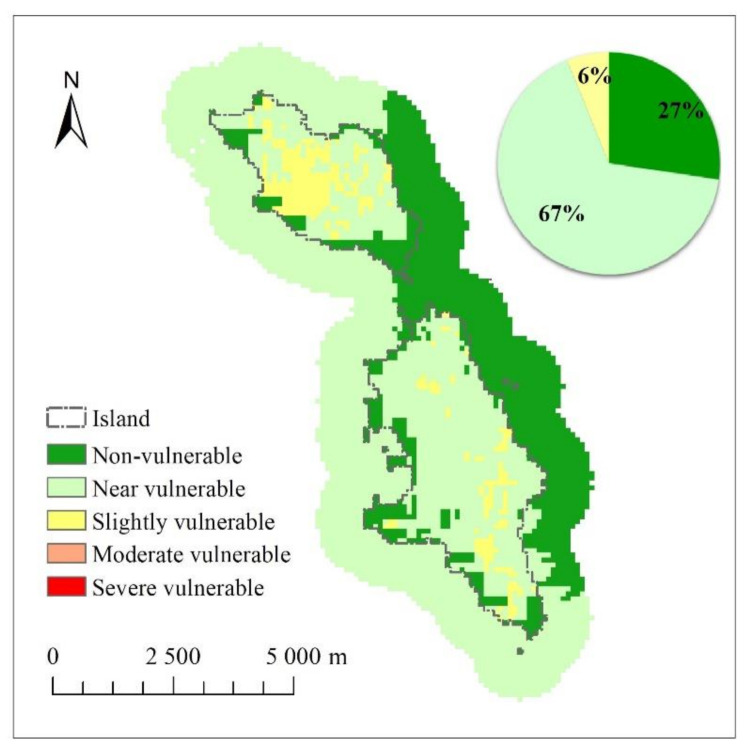
Assessment of island ecological vulnerability.

Figure 7 illustrates the integrated assessment results of ecological vulnerability of the Changshan Island. The calculation showed that the IEVI value is between 0.39 and 0.79. According to the classification of island ecological vulnerability (Table 2), the ecological vulnerability of the Changshan Island and its surrounding sea areas can be partitioned into 3 categories: non-vulnerable, near vulnerable, and slightly vulnerable area. Among them, the near vulnerable area is the largest, accounting for 67% of the study area. The ecological vulnerability of the land and sea area parts is considerably different. For the sea area, the western and eastern sea are of the Changshan Island exhibit in critical vulnerable and non-vulnerable states, respectively, indicating that the ecological vulnerability of the western sea area is higher than that of the eastern sea area. The western sea area is highly prone to ecological problems when affected by various external damaging factors such as human disturbances. Three levels of vulnerability exist in the island land area. The central and western parts of the North Changshan Island and the southeast part of the South Changshan Island are slightly vulnerable. Thus, the central and western parts of the North Changshan Island and the southeast part of the South Changshan Island are subject to relatively more external interferences, and its own attributes can be easily changed through external influences, which make the ecological environment of the region vulnerable. Most coastal areas of the island and the land area are non-vulnerable or near vulnerable. These areas are considerably affected by external interference; however, these areas are highly difficult to change because of their own attributes. Hence, ecosystem vulnerability is low. The ecological vulnerability of the North Changshan Island is higher than that of the South Changshan Island, and the ecological environment of the North Changshan Island is more vulnerable to external influence than the South Changshan Island is.

### 3.5. Conditional State Subset of the Ecological Vulnerability Index

**Figure 8:**
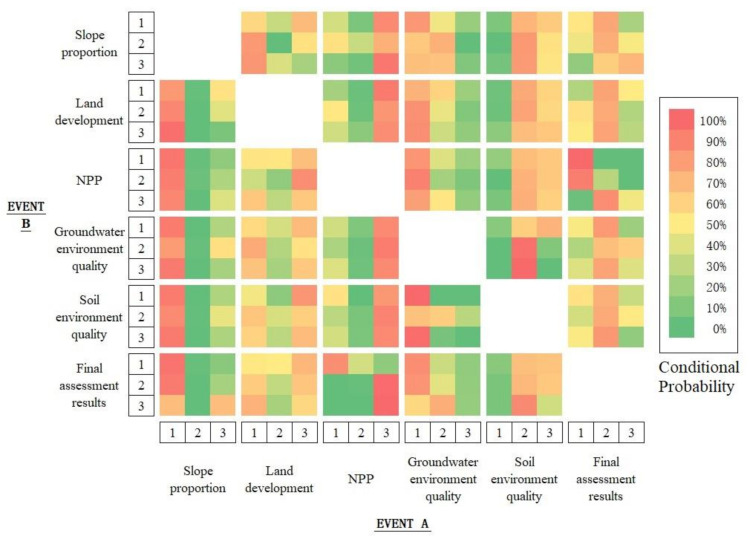
Conditional state probability of event A when event B occurs. The final assessment result and the assessment result of each indicator are divided into 3 levels individually. Except for soil environmental quality, the classification standards are according to Level 1: <0.6, Level 2: 0.6–0.7, Level 3: >0.7; soil environmental quality is classified according to Level 1: <0.24, Level 2: 0.24–0.25, Level 3: >0.25, according to the indicator evaluation results and field investigation.

In Figure 8, the color depth represents different probability conditions, and the abscissa and ordinate denote events A and B, respectively. Each color in the table represents the conditional probability P(Ai | Bj) of event A under the condition of event B. The deeper is the red color, the higher is the conditional probability; the deeper is the green color, the lower is the conditional probability.

**Figure 9:**
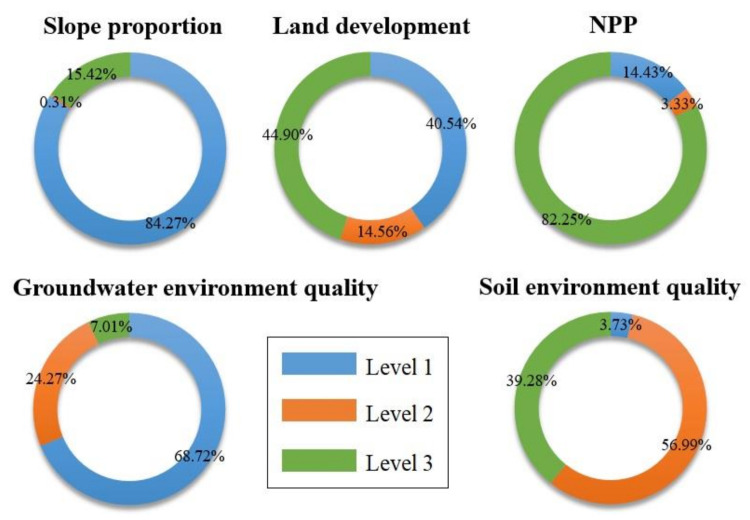
Probability of occurrence of each indicators.

According to the bottom row in Figure 8, the conditional probabilities of each index were calculated because the evaluation results are in different states. For the evaluation result of 1, the high-probability combination corresponding to each index is called the conditional state subset (CSS) 1. CSS 1 = {steep slope proportion = 1, island land development = 3, NPP = 1, groundwater = 1, soil = 2}. CSS 1 indicates that the probability of occurrence of these five indicators is the highest in the area with the ecological vulnerability of 1. Under the CSS 1 condition, the probability of occurrence of these indicators is P (assessment result = 1) = 18.52%, P (steep slope ratio = 1) = 84.27%, P (island land development = 3) = 44.90%, P (NPP = 1) = 14.43%, P (groundwater = 1) = 68.72%, and P (soil = 2) = 56.99% (Figure 9).

Similarly, for the evaluation result of 2, the high-probability combination corresponding to each index is called CSS 2. CSS 2 = {steep slope proportion = 1, island land development = 3, NPP = 3, groundwater = 1, soil = 2}. CSS 2 indicates that the probability of occurrence of these five indicators is the highest in the area with the ecological vulnerability of 2. Under the CSS 2 condition, the probability of occurrence of these indicators is P (evaluation result = 2) = 64.94%, P (steep slope ratio = 1) = 84.27%, P (island land development = 3) = 44.90%, P (NPP = 3) = 82.25%, P (groundwater = 1) = 68.72%, and P (soil = 2) = 56.99%.

Similarly, for the evaluation result of 3, the high-probability combination corresponding to each index is called CSS 3. CSS 3 = {steep slope proportion = 1, island land development = 1, NPP = 3, groundwater = 2, soil = 2}. CSS 3 indicates that the probability of occurrence of these five indicators is the highest in the region with the ecological vulnerability of 3. In CSS 3, the probability of occurrence of these indicators is P (assessment result = 3) = 16.54%, P (steep slope ratio = 1) = 84.27%, P (island land development = 1) = 40.54%, P (NPP = 3) = 82.25%, P (groundwater = 2) = 24.27%, and P (soil = 2) = 56.99%.

The aforementioned data present the highest probabilities of which index state combination should be in each ecological vulnerability assessment result grade area under the limited assessment result condition. To optimize the grade of the evaluation results, the status of the indicators must be defined. When the combination of the indicators in different states can maximize the occurrence probability of grade 1 of the evaluation results, the conditional state combination is the optimal state subset. From the rightmost column in Figure 8, the optimum state subset = {steep slope proportion = 1, island land development = 2, NPP = 1, groundwater = 1, soil = 1}. Under the optimum state subset, the probability of occurrence of these indicators is P (steep slope ratio = 1) = 84.27%, P (island land development = 2) = 14.56%, P (NPP = 1) = 14.43%, P (groundwater = 1) = 68.72%, and P (soil = 1) = 3.73%.

**Figure 10:**
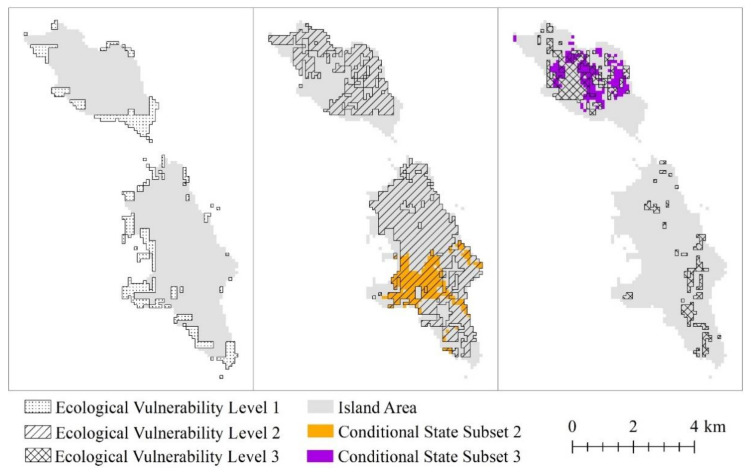
Conditional state subset.

The evaluated conditional state subsets 1, 2, and 3 and the promotable state subset are displayed in space. No regions in the study area exhibit conditional state subset 1 (Figure 10). Most regions that satisfy condition subset 2 are distributed in the middle of the South Changshan Island, and the distribution is relatively concentrated. Most regions that satisfy condition subset 3 are distributed in the North Changshan Island, and the distribution is relatively concentrated. The regional differences between the conditional state subset 2 and 3 are significant.

### 3.6. Key Indicators of Ecological Vulnerability

By using the calculation formula of the entropy difference method, we can determine whether all indexed and ecological vulnerability assessment results are marginalized to evaluate the correlation between indicators and results. Table 4 presents the entropy difference obtained for the joint occurrence of ecological vulnerability and each index and the assessment result obtained for different states (hereinafter referred to as the entropy difference degree of each index).

According to the order of entropy difference, NPP, proportion of steep slope, groundwater, soil, and island land development are ranked from high to low. The entropy difference between NPP and steep slope development is less than 1. Therefore, NPP and steep slope proportion were selected as the key indicators affecting ecological vulnerability.

## 4. Discussion

### 4.1. Promotion of Spatial Patterns

The change in land use and the promotion of spatial patterns can directly influence the vegetation cover and human activities; therefore, the promotion of spatial patterns of land use has been a sought-after topic in recent studies. The spatial promotion of land use is a means to adjust the regional land use structure in a limited area [42,43,44] for maximising the use of regional resources and achieving the pursuit of one or several purposes (economic, political, or/and ecological) [45].

By using the entropy difference method, the index with high contribution to ecological vulnerability was selected as the key index. On the basis of the key indicator selection and conditional probability table, the promotable state subset of the key variables was selected to maintain low ecological vulnerability. These key variables can be used as limiting conditions for spatial pattern promotion. The region that satisfies the promotable subset of the key indicators and that fails to reach promotable ecological conditions is defined as the suitable promotion region.

**Figure 11:**
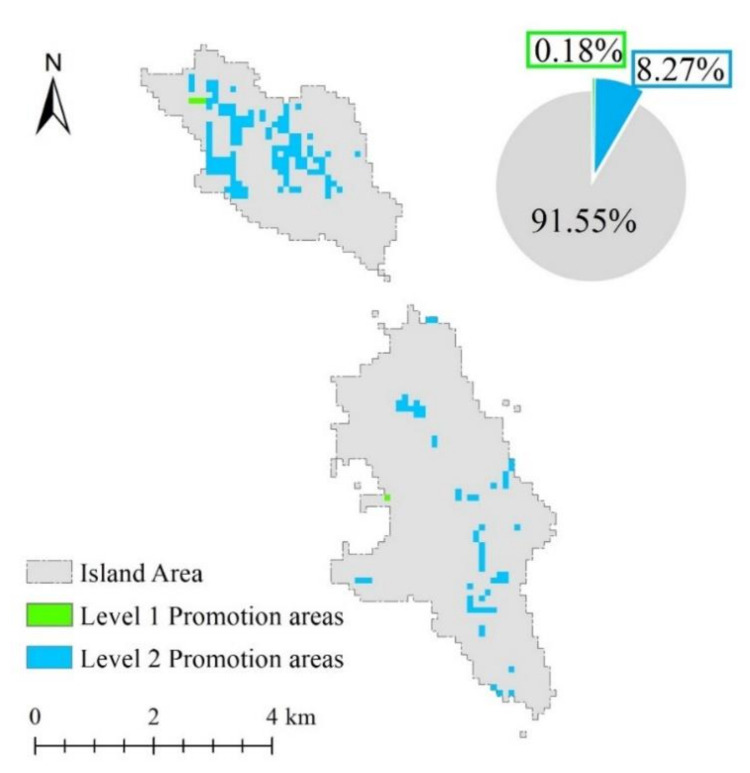
Promotable region.

From the conditional probability table, when NPP = 1 and the proportion of steep slope = 1, the probability of obtaining ecological vulnerability level 1 is the highest, and {NPP = 1, steep slope proportion = 1} is the first level state subset of the key indicators. According to the principle that the area meeting the first level state subset has a high probability of becoming the area with ecological vulnerability grade of 1, if an area satisfies the first level status subset but the assessment result is not level 1, then such an area is the first level spatial pattern promotion area, and promotion should be prioritized. Similarly, according to the conditional probability table, when NPP = 3 and the proportion of steep slope = 1, the probability of ecological vulnerability level of 2 is the highest. {NPP = 3, steep slope ratio = 1} is the secondary state subset of the key indicators. In this subset, the probability that the region becoming an area with ecological vulnerability level of 2 is the highest. According to the principle that the area meeting the second level state subset is highly likely to become the area with the ecological vulnerability level of 2, if an area satisfies the second level status subset but the assessment result grade is 3, then such an area should be a secondary spatial pattern promotion area, and this promotion level is inferior to the first level spatial pattern promotion area (Figure 11).

According to the calculation principle of the first and second level spatial pattern promotion areas, no area conformed to {NPP = 1, proportion of steep slope = 1}, and the evaluation results did not reach level 1. According to the conditional probability table, we reselected the first-level state subset of the key indicators, and set {NPP = 1 | 2, steep slope proportion = 1 | 2} as the first-level state subset of the key indicators. According to the aforementioned calculation method and spatial visualization, the first-level promotion area is considerable small, that is, only 4 hectares. Among these 4 hectares, 3 hectares and 1 hectare are located in the North and South Changshan Islands, respectively. The secondary promotion areas are mostly distributed in the North Changshan Island, and the South Changshan Island has less distribution of this secondary promotion areas. The overall area of secondary promotion is 184 hectares. Among this area, 126 and 58 hectares are present in the North and South Changshan Islands, respectively. All these areas are in the ecological vulnerability of level 3; however, according to the key factor probability, the areas are more likely to promote into ecological vulnerability of level 2. These areas should become ecological protection areas. To enhance the ecological protection of these areas and limit human development, the establishment of a relationship between the human and land must be considered.

In the first- and second-level promotion areas, the NPP and steep slope area proportion cause the areas to exhibit low ecological vulnerability. However, due to other human-made or natural influences, these areas cannot attain the optimum ecological vulnerability state. The comparison of indicators in the evaluation system indicated that the ‘island land development impact’ index of these areas is poor, that is, island land development highly influences the area that can be promoted. Therefore, during the improvement of the ecological vulnerability of these areas, local governments can reduce the development degree of these areas and control the influence of surrounding development activities on these areas to make the improved areas attain a high ecological vulnerability state.

### 4.2. Discussion and Suggestions on Ecological Red Line Area

Delimiting the marine ecological red line can provide protection to the ecological environment, ecological services, and natural resources within the red line [46]. Prohibiting and restricting the development of marine ecological red line areas can effectively ensure the ecological security of natural ecosystems and the sustainable development of human activities in the red line area [47].

In the red line scheme, six red line control areas exist in the North and South Changshan Islands and in the surrounding sea areas covered in the study, among which three are prohibited development zones and three restricted development zones. The prohibited zones include: the prohibited zone of the Changshan Island spotted seal located in the northwest of the North Changshan Island, the prohibited zone of Changshanwei geological relics situated in the South Changshan Island, and the prohibited zone of the Changshan marine park located in the north of the North Changshan Island. The restricted zones include: the restricted zone of the Changshanwei that covers the bridge between the North and South Changshan Islands, the sea area in the south of the South Changshan Island, and the restricted zone of the long island ocean park that covers the entire North Changshan Island [48].

The ecological red line area in the current red line scheme covers a large land part of the North Changshan Islands (all the land of island is classified as the prohibited or restricted development zone). However, the South Changshan Island is not covered by the ecological red line. Although this type of delimitation scheme can be used to maintain the ecological environment security of the North Changshan Island, yet it completely limits the economic and social development of the North Changshan Island. Some ecological vulnerable areas are also present in the South Changshan Island. However, the ecological red line cannot be used to protect the South Changshan Island. Although the red line ensures the economic and social development of the island, it is not conducive to the ecological protection of the island.

According to the ecological vulnerability of high-intensity development islands and the marine protection law and ecological red line delimitation opinions and rules, this study comprehensively considered the nature reserves, ecological sensitive areas, and ecological vulnerable areas located in the South and North Changshan Islands and in their surrounding sea areas and defined the regions containing these three types of areas as new ecological red line areas. The new scheme can be used to not only protect the development of key ecological functional areas but also consider the economic and social development requirements of the South Changshan Island. The new scheme can provide a scientific reference for the ecological protection of the Changshan Island and the construction of marine ecological civilisation

**Figure 12:**
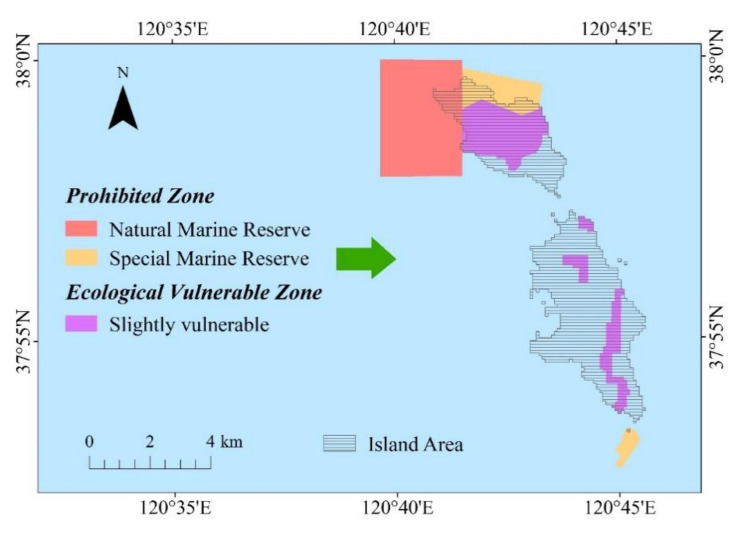
Ecological red line area.

The red area in Figure 12 presents the ecological red line delimitation of the study area based on the ecological vulnerability of the Changshan Islands and important marine ecological function areas. In the new red line area, the forbidden area for spotted seals on the Changshan Islands in the northwest of the South Changshan Island, the geological relic prohibition zone at the tail of the Changshan Island in the south of the South Changshan Island, and the forbidden area of Changshan marine park in the north of the South Changshan Island remain listed as ecological red line areas. The aforementioned areas are national marine nature reserves or special reserves, which provide important ecological services. Therefore, in addition to necessary scientific studies and ecological remediation, other human interventions and development should be prohibited in these areas. The new red line area also includes the ecological vulnerable areas of the North and South Changshan Islands based on the proposed island ecological vulnerability assessment system. Compared with the red line area in the original red line scheme, only the central part of the North Changshan Island is included in the reserve, and the southern part of the island is non-vulnerable. Therefore, appropriate establishments can be achieved to meet the regional economic development and the living requirements of residents. By contrast, during the development of the South Changshan Island, attention should be paid to the protection of the ecological vulnerable areas in the new red line area to ensure the security of the regional ecological environment.

### 4.3. Other Ecological Protection Measures and Effects

The development of most islands in China lacks scientific and reasonable planning. The economy-oriented development tends to neglect the ecological protection of islands. The island management mode, which pays substantial attention to ecological protection can lead to considerable inconvenience to the life of local residents who are limited by the geographical location of the island. The effective coordination of the ecological protection and the legitimate production and living requirements of island residents and the realization of the sustainable development of the island are the important directions of island development planning. As a comprehensive experimental area of marine ecological civilization, scientific and reasonable development planning of the Changshan Islands is crucial. Considering this study as an example, by assessing the ecological vulnerable areas of the Changshan Island, we can implement key management and control of the ecological vulnerable areas of islands and obtain reasonable development of non-vulnerable areas, which is conducive to the realization of the sustainable development goals of the islands. The social and economic development directions of the island can be utilized as starting points by highlighting advantages and improving disadvantages to achieve twice the result with half the efforts. Due to the excellent ecological environment and rich natural resources, the Changshan Islands are loved by tourists worldwide. Each year, the tourism revenue of the Changshan Islands accounts for a large proportion of the county’s fiscal revenue. Therefore, for island development, we should protect island tourism resources, the natural coastline of the Changshan Island, and other areas that can attract foreign tourists. Additionally, due to limited funds, the local government pays less attention to the science and technological development of the island. In the evaluation of the adaptability of the North and South Changshan Islands, except for the scientific study output capacity, adaptability indexes are higher than regional average levels. Therefore, we should improve the technological innovation capacity of the island, increase the financial support for the island’s scientific study programs and innovation, and reduce the risk of extensive and disordered development of the island.

The indicators selected in this study cover various influencing factors including natural factors and human interferences. Although the locations and characteristics of various islands in the world are widely different, the assessment system constructed in this study basically covers the main influencing factors that affect the ecological vulnerability of islands. Therefore, the proposed assessment system can be used to comprehensively assess the ecological vulnerability of islands. The application of the island ecological vulnerability index system can help policy makers to realise island ecological protection and rational development.

## 5. Conclusions

This study systematically investigated the human and environmental impact factors of the ecological vulnerability of the South and North Changshan Islands and their adjacent sea areas. In particular, the evaluation index system was proposed for the ecological impact of high-intensity development activities on the island, and ecological vulnerability and the key impact factors of the islands were assessed. This study can play a positive role in the ecological management of islands. The main conclusions drawn are as follows: (1) According to the results, the ecological vulnerability of the Changshan Islands is low. No area in the sea shows vulnerability, and the area near the sea is non-vulnerable and near vulnerable area. The island land area is non-vulnerable, near vulnerable, and slightly vulnerable. (2) The condition state subset can reflect the high probability combination of each indicator under various evaluation results. The promotable state subset = {steep slope proportion = 1, island land development = 2, NPP = 1, groundwater = 1, soil = 1}. In the study area, no conditional state subset 1 and first-level state subset are present. Most regions that meet condition subset 2 and 3 are distributed in the middle of the South Changshan Island and in the North Changshan Island, respectively, and their distribution is relatively concentrated. The regional differences in the two conditional state subsets are substantial. (3) The entropy difference in the NPP and proportion of steep slope is <1, indicating that the NPP and proportion of steep slope are strongly correlated with ecological vulnerability.

The study of island ecological vulnerability involves the coupling relationship of natural, social, and other systems. Although we managed to use various research methods, including GIS, field research, sampling analysis, and expert evaluation, the impact factors identified in this paper still could not completely cover all the factors that influence the ecological vulnerability. In the future studies, the indicators of island ecological vulnerability should be further refined to comprehensively assess island ecological vulnerability.

## Figures and Tables

**Table 1:** Index system for the ecological vulnerability assessment of high-intensity development islands.

Objective Layer		Element Layer	Index Layer	Index Type			Weight	Evaluation Standard	Method
Island Ecological Vulnerability	Exposure	B1 Natural pressure	C1 Impact of typical natural disasters	X	−	U	0.34	Regional mean value	Natural disaster frequency or affected area
			C2 Island area change rate	Y	−	U	0.24	Regional mean value	Comparison of multi-year area
			C3 Change rate of island shoreline	Y	−	U	0.23	Regional mean value	Comparison of multi-year shoreline length
			C4 Proportion of steep slope area	X	−	H	0.20	<1 km^2^: 0.2, 1–5 km^2^: 0.3, >5 km^2^: 0.4 (Chi et al., 2017)	Proportion of steep slope (≥15°) area
		B2 Human interference	C5 Population density of residents	Y	−	U	0.14	Regional mean value	Density of residents
			C6 Tourism population pressure	Y	−	U	0.15	Regional mean value	Density of tourism population
			C7 Impact of typical man-made environmental disturbance	Y	−	U	0.17	Regional mean value	Proportion of area affected by spilled oil and other factors
			C8 Island land development impact	X	−	H	0.20	Actual computation	Impact of land development (Appendix B.1)
			C9 Shoreline development impact	Y	−	H	0.18	Regional mean value	Proportion of artificial shoreline
			C10 Impact of surrounding sea area development	X	−	H	0.15	Actual computation	Impact of sea development (Appendix B.2)
	Sensitivity	B3 Ecological status	C11 Net primary productivity of vegetation	X	−	H	0.56	Regional mean value	Carnegie-Ames-Stanford Approach (CASA) (Appendix B.3)
			C12 Primary productivity of surrounding sea area	Y	−	H	0.44	Regional mean value	Chlorophyll content (Appendix B.4)
		B4 Environmental conditions	C13 Groundwater environmental quality	Y	−	H	0.25	Environmental quality standards	Actual measurement (Appendix B.5)
			C14 Soil environmental quality	X	−	H	0.29	Environmental quality standards	
			C15 Sea water environmental quality	X	−	H	0.28	Environmental quality standards	
			C16 Marine sediments environmental quality	Y	−	H	0.19	Environmental quality standards	
	Adaptability	B5 Self-regulation ability	C17 Island Area	X	+	U	0.54	Regional mean value	Area of island
			C18 Island shape complexity	X	−	U	0.46	Regional mean value	Length of island shoreline/[2 × (π × island area) ^0.5^]
		B6 Social support conditions	C19 Income level of residents	Y	+	U	0.28	Regional mean value	per capita disposable income
			C20 Science and technology support capacity	Y	+	U	0.37	Regional mean value	Proportion of professional personnel or marine science and technology investment
			C21 Education level of residents	Y	+	U	0.35	Regional mean value	Proportion of population with high school education or above
		B7 Environmental protection	C22 Treatment capacity of main pollutants	Y	+	U	1.00	90% (SOA, PRC, 2015)	Sewage or domestic waste treatment rate
		B8 Comprehensive management level	C23 Management effectiveness	X	+	U	1.00	Actual computation	expert evaluation

Note: According to the mandatory index, the whole index can be divided into required (X) and optional (Y) indicators; according to indicator characteristics, the whole index can be divided into positive (+) and negative (−) indicators. The larger is the positive indicator, the less vulnerable is the ecosystem; similarly, the larger is the negative indicator, the more vulnerable is the ecosystem; the spatial distribution of indicators is used to determine whether the index is the spatial unity or heterogeneity index. If an index is a spatial unity index, the same value is used to represent the index for the same time. If an index is a spatial heterogeneity index, the whole study area is assigned different values for various points for the same time.

**Table 2:** Classification of island ecological vulnerability.

IEVI	Ecological Vulnerability Level of Islands
<0.6	Non-vulnerable
0.6~0.7	Near vulnerable
0.7~0.9	Slightly vulnerable
0.9~1.0	Moderate vulnerable
>1.0	Severe vulnerable

**Table 3:** List of data.

Data Types	Data Source	Spatial Resolution	Temporal Resolution	Format	Reference Year
Socio-Economic Data	Statistics Yearbook	N/A	Yearly	Text	2011, 2016
Meteorological Data	National Meteorological Information Center, China Meteorological Administration (http://cdc.cma.gov.cn, accessed on 10 May 2020)	30 m	Daily	Raster	2016
	Resource and Environment Data Cloud Platform, Chinese Academy of Sciences (http://www.resdc.cn, accessed on 12 May 2020)				
Natural Environment	Field Investigation (Appendix A.1 and Appendix A.2)	30 m	3-Monthly	Raster	2016
	Relevant Departments of Local Government	N/A	Yearly	Text	2011–2016
Land Use/Land Cover	Resource and Environment Data Cloud Platform, Chinese Academy of Sciences (http://www.resdc.cn, accessed on 13 May 2020)	30 m	Yearly	Raster	2011–2016
Digital Elevation Model (DEM)		30 m	10-Daily	Raster	2000
Normalized Difference Vegetation Index (NDVI)	International Scientific & Technical Data Mirror Site, Computer Network Information Center, Chinese Academy of Sciences. (http://www.gscloud.cn, accessed on 13 May 2020)	500 m	Monthly	Raster	2016
Soil Data	Food and Agriculture Organization (FAO), International Institute for Applied System Analysis (IIASA)	1000 m	Multi-Year Average	Raster	2016

**Table 4:** Entropy differences in each indicator.

Indicator.	The Proportion of Steep Slope	Island Land Development	NPP	Groundwater	Soil
entropy difference	0.55	1.42	0.21	1.05	1.13

## Data Availability

Not applicable.

## Appendix A

The field investigation of the study area includes: (1) land survey, including vegetation, soil, animals, etc.; (2) adjacent sea area survey, mainly including water environment and aquatic ecology survey.

**Table A1:** List of field investigation works.

Scheme 5	Items	Number of Stations	Times	Summation
Land	Groundwater	5	4	20
Land	Soil	5	4	20
Sea	Sediment	12	2	24
Sea	Water	20	2	40

### Appendix A.1. Land Survey

#### Appendix A.1.1. Soil

5 soil samples were collected from typical plots such as farmland and woodland.

**Table A2:** Soil sample survey stations.

Sample Number	Sampling Location	
t1	N 37°56′36.588″	E 120°44′07.128″
t2	N 37°54′42.646″	E 120°44′51.321″
t3	N 37°54′55.679″	E 120°45′01.099″
t4	N 37°54′18.77″	E 120°44′24.36″
t5	N 37°50′53.832″	E 120°41′39.884″

#### Appendix A.1.2. Groundwater

According to the results of investigation, tap water has been used for drinking water in the South Changshan Island and the North Changshan Island. The natural water source is mainly distributed in several remote villages for orchard and farmland irrigation. A total of 5 wells were found. The surface groundwater was taken and the temperature, potential of hydrogen (pH) and dissolved oxygen (DO) value were measured on site. After the samples were sub packed, they were taken back to the laboratory for analysis of oil and sulphate.

**Table A3:** Underground water survey stations.

Sample Number	Sampling Location	
d1	37°56′29.571″	120°43′31.560″
d2	37°55′58.905″	120°44′21.850″
d3	37°54′45.626″	120°44′21.850″
d4	37°54′43.470″	120°44′59.094″
d5	37°54′55.596″	120°45′00.659″
d6	37°59′15.121″	120°41′41.477″

### Appendix A.2. Adjacent Sea Area Survey

Since the South Changshan Island and the North Changshan Island are centered on the extension direction of NW-SE to the main axis, the survey stations are evenly arranged according to the parallelogram. The survey area is about 15 km from east to west and 20 km from north to south. The number survey stations are S1 to S20. The suspended sediment concentration, hydrochemistry and sediment types are investigated, and 60% of the stations are selected for plankton and sediment quality investigation.

**Table A4:** Maritime investigation stations.

Scheme	Longitude	Latitude	Items		
S1	120.62197	38.00839	Water quality	Spectrum	Sediment quality
S2	120.68176	38.00911	Water quality	Spectrum	Sediment quality
S3	120.72557	38.01032	Water quality	Spectrum	Sediment quality
S4	120.77513	38.01055	Water quality	Spectrum	Sediment quality
S5	120.64075	37.95932	Water quality	Spectrum	
S6	120.70394	37.95995	Water quality	Spectrum	
S7	120.74693	37.96209	Water quality	Spectrum	
S8	120.79765	37.96293	Water quality	Spectrum	
S9	120.65847	37.92663	Water quality	Spectrum	Sediment quality
S10	120.72038	37.92827	Water quality	Spectrum	Sediment quality
S11	120.7645	37.92893	Water quality	Spectrum	Sediment quality
S12	120.81293	37.93008	Water quality	Spectrum	Sediment quality
S13	120.67766	37.89077	Water quality	Spectrum	
S14	120.73953	37.8924	Water quality	Spectrum	
S15	120.78364	37.89305	Water quality	Spectrum	
S16	120.83205	37.89419	Water quality	Spectrum	
S17	120.69646	37.8568	Water quality	Spectrum	Sediment quality
S18	120.75822	37.85812	Water quality	Spectrum	Sediment quality
S19	120.80071	37.85899	Water quality	Spectrum	Sediment quality
S20	120.84959	37.86006	Water quality	Spectrum	Sediment quality

## Appendix B

### Appendix B.1. Impact of Island Development (C8)

Impact of island development refers to the impact intensity of land development and utilization on adjacent areas. Impact of island development (LA) can be obtained by the following formula:

(A1) $$\mathrm{LA}=\overset{n}{\underset{i=1}{\sum }}LA_{i}\times IC_{i}$$

where *LA_i_* is the area of land use type *i*; *LC_i_* is the impact coefficient of the utilization type *i* on the surrounding resources and environment.

### Appendix B.2. Impact of Adjacent Sea Development (C10)

Impact of adjacent sea development refers to the impact intensity of adjacent sea development and utilization on adjacent areas. Impact of adjacent sea development (SA) can be obtained by the following formula:

(A2) $$\mathrm{SA}=\overset{n}{\underset{i=1}{\sum }}SA_{i}\times IC_{i}$$

where *SA_i_* is the area of sea use type *i*; *LC_i_* is the impact coefficient of the utilization type *i* on the surrounding resources and environment.

### Appendix B.3. Island Net Primary Productivity(NPP) (C11)

NPP refers to the total primary productivity produced by photosynthesis minus the productivity consumed by autotrophic respiration. In ecological assessment, NPP is an indicator to reflect the ecological environment of a region. CASA model is usually used for evaluation.

The model was evaluated by two factors, the Absorbed Photosynthetically Active Radiation (*APAR*) and the Actual Light Utilization Efficiency (*ε*):

(A3) $$NPP\left(x,t\right)=APAR\left(x,t\right)\times \varepsilon \left(x,t\right)$$

where *APAR*(*x*, *t*) represents the photosynthetic effective radiation absorbed by pixel *x* in month t (gC × m^−2^ × month^−1^), and *ε* (*x*, *t*) represents the actual light utilization rate (gC × mj^−1^) of pixel *x* in month *t*.

### Appendix B.4. Adjacent Sea Net Primary Productivity (C12)

The chlorophyll method is used to calculate this index. The simplified formula proposed by Cadee and Hegeman is used to calculate the index:

(A4) $$SPP=P_{s}\times E\times D/2$$

where *SPP* is the primary productivity of the surrounding sea area, *P* is the daily primary productivity of the season (mgC/m^2^ × d); *PS* is the potential productivity of phytoplankton in the surface water (within 1 m) (MGC/m2 × h), *E* is the depth of the euphotic zone (m), and *D* is the length of the daytime (h).

*Ps* can be obtained from the following equation:

(A5) $$P_{s}=C_{a}\times Q$$

where *C_a_* is the content of chlorophyll a in the surface layer (mg/m^3^), *Q* is the assimilation coefficient [mgC × (mg*Chl-a*)^−1^ × h^−1^]. The annual average *SPP* can be obtained by calculating the daily primary productivity.

### Appendix B.5. Environmental Status (C13–C16)

The environmental status indicates the level of island ecosystem pollution. In this paper, the island ecological vulnerability assessment index system includes 4 indicators: C13 groundwater, C14 soil, C15 sea water, and C16 marine sediment. The calculation method of environmental quality (*P_int_*) of each factor is as follows

(A6) $$P_{int}=\sqrt{\left[{\left(\frac{1}{n}\sum P_{i}\right)}^{2}+P_{max}{}^{2}\right]/2}$$

Among them, *P_int_* is the index of environmental quality, *n* is the number of factors, *P_i_* is the pollution index of factor *i*, and *P_max_* is the maximum value of pollution index of all factors.

*P_i_* can be obtained from the following equation:

(A7) $$P_{i}=C_{i}/S_{i}$$

where *C_i_* is the measured value of factor *i*, and *S_i_* is the standard value of factor *i*, which is evaluated according to the environmental quality standards of each environmental element.
